# Enhancement of the nutritional value of fermented corn stover as ruminant feed using the fungi *Pleurotus spp*.

**DOI:** 10.1038/s41598-021-90236-0

**Published:** 2021-06-07

**Authors:** Yuqiong Wang, Yang Luo, Lilong Luo, Hang Zhang, Yangci Liao, Changlong Gou

**Affiliations:** 1 College of Animal Science and Technology, Inner Mongolia University for Nationalities, Tongliao, China; 2 Hunan Institute of Animal and Veterinary Science, Changsha, China; 3 Institute of Pratacultural, Tibet Academy of Agricultural and Animal Husbandry Sciences, Lhasa, China

**Keywords:** Biological techniques, Biotechnology

## Abstract

Four *Pleurotus spp.* fungi (*P. diamor, P. eryngii, P. sajor-caju, P. citrinopileatus*) were compared for their potential to improve nutritional value of corn stover as ruminant feed. Corn stover was inoculated with the fungi under solid-state conditions and their results showed that *P. sajor-caju* and *P. eryngii* were better than the other two species of *Pleurotus* with respect to decreasing the acid detergent lignin (ADL) (8.99 vs 9.88 vs 10.16 vs 10.46). In contrast, *P. eryngii* had lower ability to degrade cellulose (13.38%). Corn stover treated with *P. citrinopileatus* had the highest crude protein (CP) content (7.65%), whereas treatment with *P. sajor-caju* resulted in the highest increase in essential amino acids (55.11%). Although fungal pre-treatment of lignocellulosic biomass does not always result in high-quality feed, overall, *P. eryngii* and *P. sajor-caju* improved the nutritive value of corn stover as a ruminant feed.

## Introduction

Straw, one of the most abundant agricultural wastes in China, is commonly used as a roughage source in ruminant diets. However, its high content of lignocellulosic biomass and low content of both protein and energy^1^ limit its value as a ruminant feedstuff. To improve nutritional value, various methods have been studied, including physical, chemical and biological treatments^2^. Physical methods involving extreme temperature or pressure improve palatability, but do not enhance nutritive value. Chemicals can be used to destroy cell wall structures and improve nutritive value of crop residues^3^. Although a chemical method is easy and very efficient to apply and it may result in compounds that are unsuitable for animal feed and hazardous for the environment. Biological methods, including using a fungus to degrade lignocellulosic biomass and support fungus growth, are generally more environmentally friendly than chemical pretreatment^4^. Furthermore, following fungal growth, the proteinaceous fungal mycelium also contributes to the nutritive value of solid-state fermentation materials^5^. Consequently, this treatment method can increase both digestability and protein content of straw as a source of animal feed^6^.

White rot fungi (basidiomycetous) produce various extracellular ligninolytic enzymes that delignify and break down the recalcitrant component of plant cell walls^4^. Lignin-modifying enzymes are crucial for degradation of lignin compounds for producing feed suitable for ruminants. Fungal strains vary in their ability to digest a substrate and grow, depending on substrate nutrient composition and fermentation conditions^7^. Thus, it is important to identify appropriate combinations of fungal strain and straw to optimize digestion and increase the subsequent nutritive value. The nutritional value of fermentation substrate was evaluated comprehensively, which was beneficial to promoting the straw feed process. For instance, amino acid composition and vitamin contents are important factors when evaluating feed quality. Furthermore, detailed nutritional composition of feed is important when creating a balanced ration to increase productivity and reduce production costs.

*Pleurotus spp.* is a common decomposer of agricultural residues and easy to artificially cultivate. Four representative *Pleurotus* species, namely *Pleurotus djamor*, *Pleurotus eryngii*, *Pleurotus sajor-caju*, *Pleurotus citrinopileatus* were selected due to their nontoxicity and low cost, and identify the one that resulted in the best nutrient values of corn stover (CS). For developing a solid fermentation process for the conversion of corn stover into more nutritive animal feed using *Pleurotus spp*.

## Materials and methods

### Fungal strains and spawn preparation

Four white rot fungi, *P. djamor* CGMCC 5.600, *P. eryngii* CGMCC 5.732, *P. sajor-caju* CGMCC 5.592 and *P. citrinopileatus* CGMCC 5.244 were used. They were procured from the China General Microbiological Culture Collection Center (CGMCC) in Beijing, China, grown in potato dextrose agar medium (PDA) (potato 200 g; peptone, 10 g; glucose 20 g; and agar 18 g; per L) and stored at 4 °C. Agar plates were prepared using PDA and inoculated with a 0.5 cm^2^ piece of the fungus at 25 °C for 7 d. Four agar plugs (diameter, 8 mm) of active mycelium from PDA plate was transferred aseptically into 250 ml Erlenmeyer flasks containing 80 ml of autoclaved potato extract dextrose broth medium (PDB) (potato 200 g; peptone 10 g and glucose 20 g; per L). The cultures were incubated at 25 °C in rotary shakers (150 rpm).

### Experimental set-up

Corn stover (CS) was collected from the Changling Station for Grassland and Agroecology, Chinese Academy of Sciences, Jilin, China (44° 33′ N, 123° 31′ E). The ingredients of cultivation substrate were CS (chopped to 2–3 cm lengths), corn meal 1%, urea 1%, land plaster 0.5%, K_2_HPO_4_ 1%, vitamin mix 0.5% and minerals 0.5 g/kg (comprised of Na = 0.15, K = 0.15 and Mg = 0.2). Cultivation substrate (200 g, 65% moisture) was put into an autoclavable plastic bag and sterilized at 121 °C for 1 h and allowed to cool at room temperature. Samples were inoculated with 10% spawn and incubated for 21 days in a climatic chamber at 25 ± 0.5 °C with 70–80% relative humidity. Controls were carried out in uninoculated microorganism under the same experimental conditions. Containers with inoculated CS were inoculated in triplicate.

### Chemical analyses

At 21 days after inoculation, substrate samples were dried at 60 °C until they reached a constant weight and then they were analyzed to determine nutritional value. A portion of each sample was freeze-dried, stored at − 18 °C and used to determine amino acid and vitamins contents. Total weight loss was calculated as the percentage of total solids lost after pretreatment. Total nitrogen content was determined by the Kjeldahl method, with a conversion factor of 6.25. Ethyl ether extract was determined by the Soxhlet method. The ash content was determined by ashing at 550 °C in a muffle furnace for 3 h. Neutral detergent fiber (NDF), acid detergent fiber (ADF), cellulose, hemicellulose and ADL were carried out with slightly modified method of Goering and Vansoest^8^ and Van Soest^9^. Samples (0.5–1 g) were placed into polyester mesh bags (ankom F57) and sealed. Bags and 2000 ml of neutral detergent were put into the Semi-automatic fiber analyzer (ANKOM 200i) at 100 °C for 60 min. Then, the bags were washed to neutral with distilled water, dried and weighed. Dried residue was represented as NDF. Remaining samples and 2000 ml of acid detergent were put into the Semi-automatic fiber analyzer at 100 °C for 60 min. Then, the bags were washed to neutral with distilled water, dried and weighed. Dried residue was represented as ADF. The loss was represented as hemicellulose. Dried residue was soaked in 72% (v/v) H_2_SO_4_ and kept at 25 °C for 2 h. Thereafter, the bags were washed to neutral with distilled water, dried and weighed. The loss was represented as cellulose. The remaining samples was kept at 550 °C for 3 h in a tared crucible and reweighed to calculate the loss as ADL.

### Amino acid content

An aliquot (0.2–0.5 g of each sample) was soaked in 10 ml of 6 N HCl in an autoclave at 110 °C for 24 h. The hydrolysate was filtered through a 0.22 μm cellulose acetate membrane filter before injection into the HPLC.

Amino acid composition analysis of methyl esters was done using an HPLC system (Model 1290, Agilent Technologies, Palo Alto, CA, USA) with autosampler, a Agilent Zorbax-AAA column (4.6 × 150 mm, 3.5 μm) with a Zorbax-AAA guard column (4.6 × 12.5 mm, 5 μm) and fluorescence detector. The sample was submitted to automatic precolumn derivatization with a combination of OPA reagent for primary amino acids and FMOC secondary amino acids. Mobile phase A contained 20 mmol/L natrium aceticum at pH 7.2, whereas B contained 20% antrium aceticum, 40% acetonitrile, and 40% methanol at pH 7.2. The chromatographic column temperature was set at 40 °C with a flow rate of 1 ml/minute.

### Vitamin analyses

Vitamin B1 (thiamine), B2 (riboflavin) and B6 (pyridoxine) content were assessed using HPLC. Samples (1–5 g) were extracted with 60 ml of extraction buffer (50 mg disodium ethylenediamine tetraacetic acid (Na_2_EDTA), 25 ml acetic acid, 5 ml triethylamine with deionized water added to reach a volume of 1000 ml, then 860 ml mixed with 140 ml methyl alcohol), supersound extraction for 20 min and cooled to room temperature. The sample extract was a constant volume of 100 ml. The extract was filtered through a 0.22 μm cellulose acetate membrane filter before injection into the HPLC. Analysis was carried out on Agilent Zorbax SB-C18 (4.6 × 150 mm) with Agilent Zorbax SB-C18 (4.6 × 12.5 mm) and was conducted at an excitation wavelength of 280 nm. Column temperature was 28 °C Isocratic elution with a flow of 1 ml/minute were performed using a solution of methanol and Pic-A reagent (50 mg disodium ethylenediaminetetraacetic acid (Na_2_EDTA), 1.1 g sodium heptanesulfonate, 25 ml acetic acid, 5 ml triethylamine with deionized water volume to 1000 ml).

### In vitro digestibility

In vitro digestibility (IVD) of control and treatment groups were measured according to Akthter et al.^10^, as described by Sharma and Arora^11^. Two-stage digestion included samples with fecal inoculum and acidified pepsin. Fecal inoculum was prepared by mixing fresh fecal matter (100 g/l) from cows in pre-warmed (39 °C artificial saliva) and filtered through six layers of muslin cloth. Samples (0.4 g) were placed in a 70 ml fermentation flask, with addition of 40 ml of fecal inoculums (flushing with CO_2_ gas). These fermentation flasks were kept at 39 °C for 48 h in a water bath. After fermentation ended, samples were filtered and dried at 65 °C for 48 h. Acidified pepsin (35 ml) was added to the fermentation flasks. These fermentation flasks were kept at 39 °C for 48 h in a water bath. The reaction was stopped using 100 °C water, followed by 10^4^ revolutions/minute for 20 min and then residue was filtered on a filter paper (of known weight) and dried. Weight loss in dry matter during processing was expressed as IVD.

### Statistical analyses

All data were analyzed using the General Linear Model procedure (GLM), followed by Duncan’s multiple range tests (SAS, 2008). Means were separated using least square means and presented with standard errors of the mean (SEM). The statistical model used for all data was:

$$Y_{ij} = \mu + \alpha_{i} + \varepsilon_{ij}$$

where *Y*ij = the response variable, *μ* = the general mean, *α*i = the effect of white-rot fungi and *ε*ij = the random error. Results were considered different when *P* ≤ 0.05.

## Results

### Chemical composition

In the present study, four white rot fungi significantly altered the chemical composition of CS compared to uninoculated CS (Table 1). Pretreatment of CS with any one of the four white-rot fungi increased CP 13.83–31.66% and either extract (*P* < 0.01), but concurrently reduced NDF, ADL, hemicellulose (*P* < 0.001), ADF and cellulose (*P* < 0.01) content of CS. Treatment with *P. citrinopileatus* resulted in the greatest increase (*P* < 0.05) in CP content, whereas the greatest increase in EE was in CS exposed to *P. diamor,* followed by *P. citrinopileatus*, *P. sajor-caju* and *P. eryngii*. Furthermore, all four white rot fungi caused net reductions in DM, OM, NDF, ADF, ADL, cellulose and hemicellulose (Table 2). The loss of cell wall constituents was smallest (*P* < 0.05) for *P. eryngii* and highest for *P. diamor*. Corn stover pre-treated with *P. diamor* had the greatest reduction in NDF (30.74%) and ADF (25.79%), whereas *P. sajor-caju* degraded maximum ADL (40.95%), followed by *P. citrinopileatus*; this fungus also caused maximum degradation of hemicellulose (31.44%) and cellulose (26.62%). *P. eryngii* increased maximum CP (31.66%) but caused the least degradation of DM (8.41%). All fungi resulted in significantly higher IVD compared to the autoclaved CS. Incubations with *P. sajor-caju* resulted in the highest IVD (118.69%) followed by *P. eryngii,* whereas *P. citrinopileatus* had the least change in IVD (18.18%).

**Table 1:** Chemical composition of corn stover after 21 days of incubation with various white rot fungi (*Pleurotus* sp.) or control.

Fungi/sample	*P. diamor*	*P. eryngii*	*P. sajor-caju*	*P. citrinopileatus*	Autoclaved straw (control)	SEM	*P* value
CP	7.11^ab^	6.84^b^	6.70^b^	7.65^a^	4.75^c^	0.208	< 0.001
Ash	9.93^a^	9.35^a^	9.55^a^	9.56^a^	2.61^b^	0.214	< 0.001
EE	1.36^a^	1.04^b^	1.22^ab^	1.23^ab^	0.75^c^	0.08	0.003
NDF	63.95^b^	63.64^bc^	65.11^b^	62.15^c^	68.76^a^	0.52	< 0.001
ADF	48.57^b^	49.36^b^	48.34^b^	48.63^b^	51.56^a^	0.384	< 0.001
ADL	10.46^b^	9.88^bc^	8.99^c^	10.16^b^	12.31^a^	0.315	< 0.001
Hemicellulose	29.1^bc^	29.31^b^	28.69^bc^	27.84^c^	32.24^a^	0.383	< 0.001
Cellulose	47.55^b^	48.07^b^	48.35^b^	46.97^b^	50.82^a^	0.575	0.007
IVD %	40.58^c^	44.05^b^	46.56^a^	25.16^d^	21.29^e^	0.225	< 0.001

SEM: standard error of mean.

^a–e^Within a row, means without a common superscript differed (*P* < 0.001).

**Table 2:** Loss of nutrients (%) from corn stover after 21 days of incubation with various white rot fungi (*Pleurotus sp*.) or control.

Fungi/sample	*P. diamor*	*P. eryngii*	*P. sajor-caju*	*P. citrinopileatus*	SEM	*P* value
DM	21.2^a^	8.41^b^	19.2^a^	20.63^a^	0.708	< 0.001
CP	− 17.81^a^	− 31.66^b^	− 13.83^a^	− 27.7^b^	1.75	< 0.001
NDF	30.74^a^	16.44^c^	25.62^b^	28.28^ab^	0.946	< 0.001
ADF	25.79^a^	12.33^b^	24.25^a^	25.15^a^	0.71	< 0.001
ADL	33.03^ab^	26.5^b^	40.95^a^	34.44^ab^	2.542	0.025
Hemicellulose	28.87^a^	16.74^b^	28.09^a^	31.44^a^	1.29	< 0.001
Cellulose	26.29^a^	13.38^b^	23.13^a^	26.62^a^	1.22	< 0.001

SEM: standard error of mean.

^a–b^Within a row, means without a common superscript differed (*P* < 0.001).

### Amino acids

Amino acids are secondary metabolites of fungi and reliable indicators of nutritional value^12^. There were differences among strains (*P* < 0.05) in how white-rot fungi CS affected amino acid content (Table 3). After 21 days of incubation, there were increases in content of most amino acids, expect for alanine (Ala), methionine (Met), tyrosine (Tyr). Incubation of CS with fungi increased leucine (Leu), phenylalanine (Phe), lysine (Lys) (*P* < 0.001), threonine (Thr) (*P* < 0.01) and valine (Val) (*P* < 0.05) compared to the control. Compared to autoclaved straw, Leu was the most abundant (48.27, 25.46, 49.33 and 54.4% in *diamor*, *eryngii*, *sajor-caju*, *citrinopileatus*, respectively). Val was the second most abundant essential amino acid, followed by Lys, Thr, Phe, Met., Incubation of CS with *sajor-caju* and *citrinopileatus* resulted in the largest increases in essential amino acids. Maximum increases in Thr, Val and Met occurred in CS incubated with *sajor-caju*, wherereas *citrinopileatus* maximized Try, Phe, Leu and Lys. Regarding non-essential amino acids, *diamor* maximized cysteine (Cys) and histidine (His), whereas *eryngii* maximized arginine (Arg) and Glu was maximized by *sajor-caju*. Maximum of Ala, Asp, Gly, Ile, Ser and Tyr were increased by *citrinopileatus* incubation of CS, but Ala and Tyr were not significantly different compared to other treatment groups.

**Table 3:** Amino acid composition (mg/g) of corn stover after 21 days of incubation with various white rot fungi (*Pleurotus* sp.) or control.

Item	*P. diamor*	*P. eryngii*	*P. sajor-caju*	*P. citrinopileatus*	Autoclaved straw (control)	SEM	*P* value
Leu*	16.77^a^	14.19^b^	16.89^a^	17.47^a^	11.31^c^	0.478	< 0.001
Lys*	14.82^ab^	12.32^b^	13.58^bc^	15.78^a^	7.37^c^	0.44	< 0.001
Met*	0.87^b^	1.77^ab^	2.63^a^	1.12^b^	1.95^ab^	0.422	0.089
Phe*	9.84^a^	8.57^b^	9.93^a^	10.02^a^	6.57^c^	0.37	< 0.001
Thr*	12.17^ab^	10.62^bc^	12.71^a^	12.62^a^	8.96^c^	0.549	0.003
Val*	14.96^ab^	13.33^b^	16.62^a^	14.97^ab^	10.49^c^	0.831	0.004
Ala	20.66^a^	18.37^a^	20.1^a^	21.3^a^	18.56^a^	1.55	0.615
Arg	16.77^b^	20.44^a^	18.07^ab^	19.54^b^	5.33^c^	0.92	< 0.001
Asp	24.96^a^	21.55^b^	26.5^a^	26.57^a^	14.42^c^	0.849	< .001
Cys	6.03^a^	5.7^a^	5.96^a^	5.8^a^	1.92^b^	0.409	< 0.001
Glu	27.98^a^	29.2^a^	30.82^a^	27.98^a^	22.99^b^	1.07	0.005
Gly	13.72^ab^	12.34^b^	13.8^b^	14.26^a^	9.27^c^	0.451	< 0.001
His	4.62^a^	4.22^a^	4.31^a^	4.24^a^	1.89^b^	0.38	0.003
Ile	10.68^a^	9.04^b^	10.86^a^	11.03^a^	7.08^c^	0.386	< 0.001
Ser	11.63^a^	10.41^a^	11.8^a^	12.01^a^	7.43^b^	0.508	< 0.001
Tyr	2.79^a^	1.97^a^	2.41^a^	2.84^a^	2.56^a^	0.29	0.288

*Means essential amino acid.

SEM: standard error of mean.

^a–c^Within a row, means without a common superscript differed (*P* < 0.001).

### Vitamins

After 21 days of fermentation, there were significant differences among fungi in content of thiamine, riboflavin, pyridoxine, folic acid, niacin and vitamin C (Table 4). Compared to the control group, thiamine (B1) contents of *diamor* and *sajor-caju* treatment groups were higher, whereas *eryngii* and *citrinopoleatus* were lower. Thiamine was highest in the *diamor* group, but lowest in the *citrinopoleatus* group. Incubation with *sajor-caju* resulted in the greatest increase (*P* < 0.001) of pyridoxine (B6), whereas *eryngii* had the least. Incubation with *sajor-caju* had the highest (*P* < 0.001) folic acid, whereas this vitamin was not detected after incubation with *citrinopileatus* or in autoclaved straw. All four white rot-fungi increased (*P* < 0.001) niacin contents of CS, whereas it was highest with *diamor* (*P* < 0.001). All treatments increased (*P* < 0.001) vitamin C, except for *citrinopileatus*. Incubation of substrates with *sajor-caju* maximized vitamin C content.

**Table 4:** Vitamin composition (mg/kg) of corn stover after 21 days of incubation with various white rot fungi (*Pleurotus* sp.) or control.

Fungi/sample	*P. diamor*	*P. eryngii*	*P. sajor-caju*	*P. citrinopileatus*	Autoclaved straw (control)	SEM	*P* value
B1 (thiamine)	1.64^a^	0.61^c^	1.55^a^	0.5^c^	0.85^b^	0.076	< 0.001
B2 (riboflavin)	0.57^b^	0.57^b^	0.28^c^	0.29^c^	0.92^a^	0.049	< 0.001
B6 (pyridoxine)	0.97^b^	0.46^c^	1.32^a^	1.02^b^	0.97^b^	0.074	< 0.001
Folic acid	27.25^d^	250.03^b^	327.41^a^	61.37^c^	0^e^	1.92	< 0.001
Niacin	31.28^a^	22.53^c^	24.76^b^	18.05^d^	0.025^e^	0.257	< 0.001
VC	19.03^c^	22.08^b^	24.91^a^	0^d^	0^d^	0.226	< 0.001

SEM: standard error of mean.

^a–d^Within a row, means without a common superscript differed (*P* < 0.001).

## Discussion

Changes in chemical composition of crop residues induced by fungi have been reported^13,14^. Growth of fungal mycelium was supported by degradation of lignocelluloses, increasing total protein content in the fermentation substrate and improving its nutrition quality. Similarly, in another report, *P. diamor* increased CP more than other fungi^12^. Differences among fungi in protein content after fermentation was related to the increase of fungal biomass. Furthermore, *P. sajor-caju* had a strong ability to degrade ADL. Lignin degradation of straw was positively correlated with IVD^15^. Thus, IVD was increased during solid state fermentation of agricultural residues by *P. sajor-caju*, similar to the results of this experiment. Therefore, we presumed *P. sajor-caju* had higher selectivity to lignin degradation compared to cellulose and hemicellulose. In addition, digestibility is also related to fermentation time and constituents, concentration and structures of various plant cell wall polymers^16^.

Differences among substrates in their biological and chemical properties greatly affects nutritional value of the fermentation substrate. Biodelignification of wheat straw by solid state fermentation with white-rot fungi has been reported^17,18^. However, there are limited data regarding chemical composition of CS treated with white-rot fungi. In general, most substrate studies have used locally available agriculture wastes. In China, much of the CS is burned or disposed of in the field. Since not much CS is used for animal feed, it was chosen as a fermentation substrate for this study. To reduce fermentation time, we used liquid spawn fermentation of CS to increase protein content, reduce organic matter losses and shorten fermentation interval to produce feed suitable for ruminants. Nutritional values of substrates were directly associated with duration of incubation^19^, with reductions in lignin content and increases in CP content. It is noteworthy that reductions in cellulose and hemicellulose that occurred in the present study are not essential to enhance feed value for ruminants, as these livestock have the ability to degrade and utilize these substances.

It is well known that essential amino acids must be ingested from the diet and therefore must be considered in diet formulation. Analyzing amino acid composition of feedstuffs ensures that nutritional needs are met^20^ and supplementing essential amino acids may increase efficiency of animal production and enable low-cost ration formulation^18^. In this experiment, fermentation substrate had high concentrations (increased by 1.25–2.14 times compared to the control) of essential amino acids. Similar to the present observation, the amino acids profile of paddy straw was improved by less than a factor of two by incubation with *Crinipellis sp*. RCK-1 for 5 days at 30 ± 2 °C ^21^. Remarkably, exposure of paddy straw to *Pleurotus ostreatus* for 20 days improved amino acid content by as much as 15 times^22^, much higher than the results of present study. Differences among studies are dependent on fungal species, fermentation substrate and fermentation time, as well as control component.

In comparisons of fungal fermentation of straw and alfalfa and orchardgrass^23^ amino acid contents of the straw were highest, highlighting potential to replace roughage as protein source for ruminants, especially where feed resources are limited In the present study, content of essential amino acids was higher in the fermentation substrate of *sajor-caju* and *citrinopileatus* after 21 days. The content of essential amino acids in the CS fermented by sajor-caju and citrinopileatus were compared to common feed material (Table 5). Although five essential amino acids in the fermentation substrate were lower than insoybean meal, they were 1.87–6.46 times higher than in wheat bran and maize meal. Therefore, fermented CS is valuable as a source of amino acids.

**Table 5:** Content (mg/kg) of essential amino acids in corn stover after 21 days of incubation with two white rot fungi (*P.* or *P*. *citrinopileatus*) compared to common feeds.

Item	*P. sajor-caju*	*P. citrinopileatus*	Soybean meal	Wheat bran	Maize meal
Leu	16.89	17.47	27.54	9.44	9.35
Lys	13.58	15.78	17.96	6.08	2.44
Phe	9.93	10.02	19.45	6.71	3.88
Thr	12.71	12.62	14.27	4.99	2.45
Val	16.62	14.97	17.06	7.67	4.08
Sulfur-containing amino acids	8.59	6.92	8.88	4.22	3.57

The *Plearotus spp*. are not only rich in essential amino acids, but also in vitamins^24^. Vitamins have important functions in animals, including essential metabolism^25^. It is noteworthy that the vitamin content after fermentation varies widely among fungal species^26^. It is interesting that riboflavin (B2) contents were 43.49–69.56% than lower than the control group in this experiment, apparently due to utilization by fungi. However, B1 content was increased, particularly by *sajor-caju*. Dietary requirements for many vitamins in ruminants are poorly defined. It has long been believed that the amount of B vitamins synthesized by rumen microorganisms can meet the nutritional requirements of animals. For adult ruminants, the sources of vitamin B are mainly from the synthesis of rumen microorganisms, so that supplementation of vitamin B are not required in the ruminant diet under normal circumstances. However, the NRC^27^ recommends that milk replacers for calves should include B1, B2, B6, niacin and folic acid at 6.5 mg/kg DM, 6.5 mg/kg DM, 0.1 mg/kg DM, 10 mg/kg DM and 0.5 mg/kg DM, respectively. Some studies have shown that vitamin B supplements in the diet have many benefits for ruminants. For example, B vitamins stimulate cellulose bacteria in the rumen and they improve the digestibility of cellulose during in vitro fermentation^28^. Although daily niacin supplementation increased milk protein for cows in early lactation, it is not normally used in the beef industry^29^. Folic acid deficiency causes megablastic anemia and affects fetal development during pregnancy^30^. Accordingly, supplementing B vitamins in ruminant diet is related to animal species, diet and animal production stage. Vitamin C is an indispensable antioxidant component of animal feed, with important roles in animal health and immune function^31^. Dietary supplementation with vitamin C increases the concentration of vitamin C in skeletal muscle^32^. This will be promote stability of oxymyoglobin and lipid, which results in maintaining meat quality. Vitamin C supplementation in ruminants can have beneficial effects, especially under conditions of environmental stress. Furthermore, vitamin C supplementation in sheep can effectively relieve the stress of water shortage^33^. Additionally, vitamin C supplementation improved the tenderness of the beef longissimus dorsi and the fatty acid profile of meat products^34^.

## Conclusions

These four white rot fungi had high selectivity for lignin and increased CP content and IVD. In detail, *eryngii* fungi had the lowest reduction in dry matter during 21 days of incubation, whereas *eryngii* and *sajor-caju* fungi maximized lignin degradation and retention of cellulose, thereby improving rumen fermentability. In addition, incubation of substrates with these fungi improved contents of specific amino acids and vitamins. Therefore, these fungi have potential to improve the nutritional value of CS as a ruminant feed, with *P. eryngii* and *P. sajor-caju* yielding the best outcomes.
